# Tribo‐Induced Color Tuner toward Smart Lighting and Self‐Powered Wireless Sensing

**DOI:** 10.1002/advs.202004970

**Published:** 2021-05-02

**Authors:** Jiaqi Wang, Haoyu Wang, Kedong Yin, Yunlong Zi

**Affiliations:** ^1^ School of Marine Sciences Sun Yat‐Sen University Zhuhai 519082 China; ^2^ Department of Mechanical and Automation Engineering The Chinese University of Hong Kong Shatin, N.T. Hong Kong China; ^3^ Southern Marine Science and Engineering Guangdong Laboratory (Zhuhai) Zhuhai 519080 China

**Keywords:** self‐powered systems, smart lighting, triboelectric nanogenerators, underwater photographing, wireless sensing

## Abstract

The color‐tuning capability of solid‐state lighting (SSL) systems are highly demanded for smart lighting according to the environmental conditions, as well as wireless sensing of the environmental information. In the meanwhile, state‐of‐the‐art triboelectric nanogenerator (TENG)‐based sensing systems rely on bulky and expensive devices, which require cable connections and additional power consumptions. This work aims at solving these challenges, through developing a tribo‐induced color tuner that can be integrated into the vastly distributed commercial SSL system. This tribo‐induced color tuner includes a concentric color conversion plate consisting of (Sr,Ca)AlSiN_3_:Eu phosphor and TiO_2_, a tribo‐induced liquid lens, and a rotary freestanding sliding TENG. The color oscillation between purple and pink is achieved upon the tribo‐charging by the TENG, which reveals the input mechanical motion signals. The signal can be conveniently sent by everywhere‐existed lamps and processed by everyone‐owned smartphone cameras or closed‐circuit televisions. Through this approach, the function of wireless sensing is achieved without the need of preamplification, with no additional power supply required, as demonstrated for wireless sensing of the rotation speed. The smart lighting for underwater photographing is also demonstrated by the color‐tunable SSL system with the best imaging quality achieved.

## Graphical abstract

A tribo‐induced color tuner is developed through integrating a triboelectric nanogenerator, a concentric color‐conversion plate, and a liquid lens, which can be incorporated into solid‐state lighting systems. The tribo‐charging can tune the color of the illumination system, benefiting the color‐tunable smart lighting (i.e., underwater photographing) and wireless sensing (i.e., wind speed monitoring).

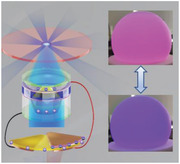

## 1 Introduction

Nowadays, solid‐state lighting (SSL) systems are widely applied in the modern society ranging from indoor to outdoor lighting applications, replacing conventional incandescent and fluorescent lamps owing to their high‐efficiency and environmental friendly characteristics.^[^ ^1^, ^2^, ^3^, ^4^, ^5^, ^6^, ^7^ ^]^ It has been estimated that the market size of the SSL is to exceed the demand of 10 billion units by 2024.^[^ ^8^ ^]^ The output spectrum of the SSL device is usually achieved by the spectral superposition of the fluorescent light from the phosphor and diode‐emitting light.^[^ ^9^, ^10^, ^11^, ^12^ ^]^ However, until now, the output spectrum of the SSL device cannot be tuned once packaged, while it is desirable to tune the illumination spectrum according to different conditions in some circumstances toward smart lighting. For example, the tunable illumination spectrum of the underwater lighting source is desired depending on the normal or red‐tide seawater conditions toward the optimized photographing quality.^[^ ^13^, ^14^, ^15^, ^16^ ^]^ For the outdoor illumination, the yellowish light has a long penetration distance at the foggy condition;^[^ ^17^, ^18^ ^]^ however, it is not preferred in normal conditions. And for the indoor SSL systems in the building, it is preferred to present different correlated color temperatures (CCTs) according to different activities.^[^ ^19^, ^20^, ^21^ ^]^

Furthermore, the color of the SSL can be utilized as the information carrier, wirelessly transmitting the information through the color oscillation frequency, color‐shift displacement and so on. If the color tuning can be triggered by the environmental factors such as winds, vibrations, ocean waves, the wireless sensing can be achieved which is in urgent demand with the rapid development of the Internet of Things (IoTs). The time‐variant color emitted by SSLs can be monitored by a camera serving as the receiver in a cost‐effective way. Thanks to the well‐developed and vastly distributed closed‐circuit television (CCTV) and the smartphone owned by almost everyone, the camera is everywhere available in our daily lives, which means the extra receiver is not required for such the color‐tuner‐based wireless sensing system. However, how to enable the color tuning of the SSL by the environmental information is still a challenging issue, which requires the assistance of energy harvesters.

Triboelectric nanogenerator (TENG), first invented in 2012, is regarded as a promising energy harvester which scavenges ubiquitous mechanical energies from ambient environments through the coupling effect of triboelectrification and electrostatic induction,^[^ ^22^, ^23^, ^24^, ^25^, ^26^ ^]^ enabling self‐powered systems.^[^ ^27^, ^28^, ^29^, ^30^, ^31^ ^]^ TENGs are not only energy harvesters, but also active sensors with high signal‐to‐noise ratios (SNRs), revealing the mechanical motion profiles through their output electrical signals,^[^ ^32^ ^]^ which have been successfully employed to measure the wind speeds,^[^ ^33^ ^]^ structural vibrations,^[^ ^34^ ^]^ ocean wave oscillations,^[^ ^35^ ^]^ heart rates,^[^ ^36^ ^]^ displacements,^[^ ^37^ ^]^ biomechanical motions,^[^ ^38^ ^]^ etc. Since the charge output from the TENG is extremely low bringing difficulties to process the signal, a precise electrometer embedded with a preamplifier is usually necessary to characterize the time‐variant charge transfer.^[^ ^32^, ^35^, ^39^, ^40^, ^41^ ^]^ However, such the electrometer is usually bulky and expensive, and requires cable connections, which is not preferred or even not allowed in some harsh circumstances.^[^ ^42^, ^43^ ^]^ Besides, through this traditional method, a truly self‐powered sensing system can be hardly achieved, since the required devices and circuits for signal processing and power management consume additional power, which is usually larger than the energy provided by the TENG.^[^ ^32^ ^]^ Alternative solutions are urgently required to achieve TENG‐based self‐powered sensing systems.

In this work, a tribo‐induced self‐powered color tuner was developed by the conjunction of a liquid lens (LL) assisted phosphor converter and a rotary freestanding sliding TENG (RFS‐TENG). Upon tribo‐charging of the LL by the TENG, the focal length variation induced the change of the beam spot size on the phosphor plate, shifting the emission spectrum and hence the color. The developed color tuner was successfully incorporated into a commercial SSL system to achieve smart lighting and wireless sensing applications. A seawater‐condition dependent smart lighting system was demonstrated for obtaining the best underwater photographing quality. To achieve self‐powered wireless sensing, the camera served as the receiver to remotely monitor the mechanical signal. Compared with the conventional TENG‐enabled self‐powered sensors,^[^ ^35^, ^39^, ^40^, ^41^ ^]^ the signal can be sent wirelessly by everywhere‐existed lamps and processed by everyone‐owned cameras or CCTVs, without the cable connection and high energy consumption brought by additional circuits and devices.

## 2 Results

**Figure 1:**
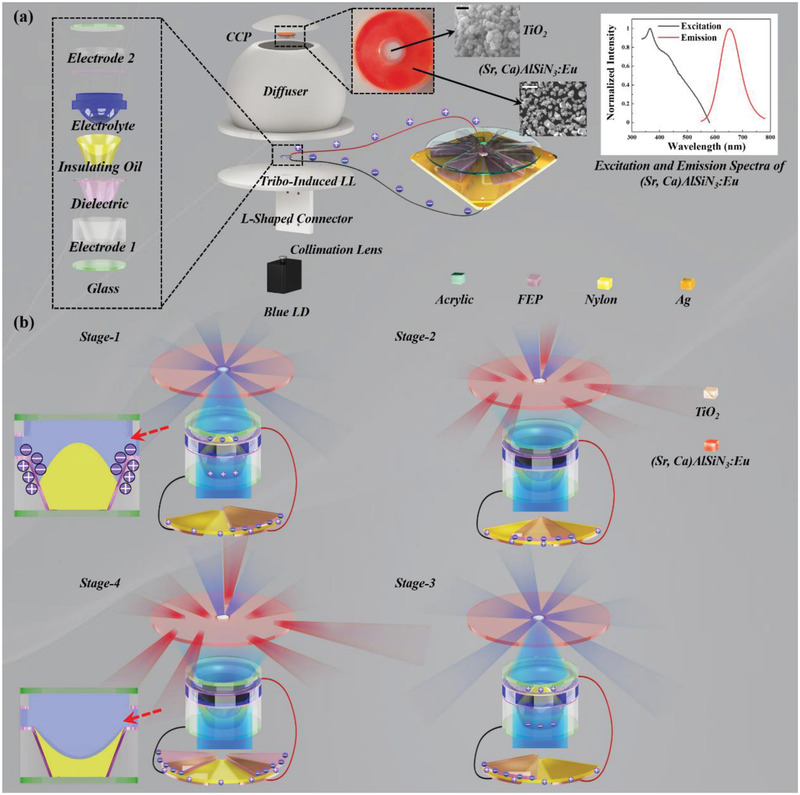
The structure and operation mechanism of the tribo‐induced self‐powered color tuner. a) Schematic diagram of the developed tribo‐induced self‐powered color tuner as integrated with the SSL system. The color tuner consists of a tribo‐induced LL connecting with the RFS‐TENG and a CCP. Through the assistant of L‐shaped connector, the color tuner can be physically connected with the blue LD and 3D printed diffuser. The left side of the figure illustrates the decomposed parts of the tribo‐induced LL. The two insets at the top center illustrates the SEM photographs of the TiO_2_ (scale bar: 100 nm) and (Sr, Ca)AlSiN_3_:Eu (scale bar: 50 *μ*m). Besides, the relative excitation and emission spectra of the (Sr, Ca)AlSiN3:Eu phosphor is illustrated in the right top corner. b) The electrical and optical behavior of the developed color tuner incorporated in the SSL system in one electrical cycle. The optical oscillation frequency of the developed color tuner is twice of the electrical frequency of the RFS‐TENG. The left‐sided two insets illustrate the cross‐sectional diagram of the LL with and without tribo‐charging at stages 1 and 4.

As shown in **Figure** **1**a, a tribo‐induced self‐powered color tuner was developed, which can be easily incorporated into an SSL system. The tribo‐induced color tuner consists of an RFS‐TENG, a LL (Dow‐Corning Varioptic Ltd) and a concentric color‐conversion plate (CCP). The RFS‐TENG was utilized to scavenge the mechanical energies such as wind, water flow and wheel rotation from ambient environments to charge the LL in alternating current (AC) mode. The operation of the RFS‐TENG is based on the coupling effect of triboelectrification and electrostatic induction, resulting in the voltage drop on the LL. The focal length of the LL varies when different voltage biases are applied. The working mechanism of the LL is based on the electrowetting effect, where the interfacial tension between conductive and insulative liquids is changed when the electric field is loaded.^[^ ^44^, ^45^, ^46^ ^]^ Our previous work has investigated the TENG triggered electrowetting effect.^[^ ^44^ ^]^ In the LL, a conductive electrolyte and an insulative dielectric oil, were employed to form the tunable interface. The refractive indices (RIs) of the electrolyte and the dielectric oil are 1.500 and 1.438 at the wavelength of 450 nm. As shown on the left side of Figure 1a, the decomposed illustration of the employed LL is indicated. When the LL is not charged, the dielectric oil presents a concave shape governed by the interfacial tension equilibrium among the dielectric oil, electrolyte and the dielectric coated layer. As a result, the incident light is diverged by the LL. When the LL was tribo‐charged, the electrolyte suffered from the electrostatic force induced by the electric field between two electrodes to form the convex shape, and thus the incident light was focused, as illustrated in Figure S1 (Supporting Information) in the supporting information. Given the illumination position in a certain distance from the lighting source, the illuminated beam spots were different when different voltage drops were loaded on the LL.

The CCP was placed 10 cm above the LL, which is able to partially convert the incident blue photons into the fluorescent photons. The CCP was fabricated with two concentric layers, the (Sr,Ca)AlSiN_3_:Eu phosphor and TiO_2_ with resin matrix, respectively, with the fabrication process detailed in the Method Section. The diameters of the (Sr,Ca)AlSiN_3_:Eu and TiO_2_ encapsulation layers are 4.8 and 21 mm, respectively, making the area of the (Sr,Ca)AlSiN_3_:Eu 16.95 times of that of TiO_2_. The scanning electron microscope (SEM) photographs of the (Sr,Ca)AlSiN_3_:Eu and TiO_2_ particles are shown as insets in Figure 1a. It can be identified that these two types of particles were with mean particle diameters of around 15 *μ*m and 50 nm, respectively. The RIs of (Sr,Ca)AlSiN_3_:Eu, TiO_2_ and resin binder are 1.91, 2.78, and 1.5, respectively, as shown in Table S1 in the Supporting Information. For the two concentric layers, the weight mixing ratios are 0.3:1 for (Sr,Ca)AlSiN_3_:Eu:resin and 0.05:1 for TiO_2_:resin. With such high mixing ratios, the phosphor presented the strong absorption effect while the TiO_2_ performed strong scattering capability. The (Sr,Ca)AlSiN_3_:Eu absorbed the blue photons emitted from the blue LDs and re‐emitted fluorescent photons, with the excitation and emission spectra shown on the right top corner of Figure 1a. It can be identified that the fluorescent emission light peaks at the 650 nm wavelength. Besides the absorption and fluorescent re‐emission performance of the (Sr,Ca)AlSiN_3_:Eu, such a microsized particle cluster also presents a multiple‐scattering capability owing to the RI difference between the (Sr,Ca)AlSiN_3_:Eu and resin binder. The TiO_2_ only serves as the scatterer. According to the Lorenz–Mie theory,^[^ ^47^, ^48^, ^49^ ^]^ TiO_2_ particles present much higher scattering capability compared to the (Sr,Ca)AlSiN_3_:Eu due to its smaller size and larger RI contrast, with calculations performed to characterize the angular scattering pattern of the phosphor and TiO_2_, as indicated in the Figure S2 in the Supporting Information. A commercial LD, Nichia BDB7875, was chosen in this study, serving as a high‐intensity SSL device, providing blue source photons.^[^ ^50^ ^]^ The threshold injection current for operation is 80 mA, and the maximal allowed injection current should be below 1.7 A. Depending on the current loading and thermal dissipation conditions, the resulted voltage drop on the LD ranges from 3 to 6 V. The LL, CCP and blue LD were mechanically connected, as described in the method section. And a 3D printed ball‐shaped diffuser was screwed above the LL, where the CCP was centered at the top of the diffuser, which randomized the photons and facilitated the color mixing to achieve the uniform illumination.

The output spectrum of the developed SSL system was achieved by the spectral superposition of the (Sr,Ca)AlSiN_3_:Eu re‐emitted fluorescent light and transmitted blue photons. When the RFS‐TENG was not triggered, the LL was regarded as a concave lens with zero voltage drop. At this moment, the beam spot is greatly enlarged, illuminating the whole phosphor plate covering both (Sr,Ca)AlSiN_3_:Eu phosphor and TiO_2_, leading to the pink light illumination since a great proportion of blue photons were converted into red photons. When the RFS‐TENG was triggered, the shape of the dielectric oil was changed from concave to convex. As a result, the light passing through the LL was focused on the TiO_2_ zone, resulting in the majority of blue photons being scattered by the TiO_2_ and minority of blue photons being converted into the red photons by the phosphor, forming a purple color. By doing so, the color of the SSL device can be effectively tuned through the tribo‐charging triggered by the RFS‐TENG. By connecting with an external capacitor to the color tuner for lowering the voltage drop on the LL, the desired output color spectrum can be effectively selected from a broad range between the pink state (TENG off) and the purple state (TENG on). Even though in the majority of cases, human eyes cannot detect the blinking of the developed color‐tunable SSL device due to high frequency of signals, cameras embedded in the smartphones or CCTV available everywhere are able to monitor the time‐variant color performance, which can reflect the profile of the environmental mechanical stimuli.

The structural design of the developed RFS‐TENG was illustrated in Figure 1a, where a bent fluorinated ethylene propylene (FEP) film embedded in an acrylic plate and a nylon film above the Ag coated printed circuit board (PCB) functioned as the rotator and stator for the triboelectrification, respectively. The contact between two triboelectric layers was supported by the bending‐induced elastic force of the FEP. Such the soft, deformable substrates gently contact together which minimized the friction wear loss during the operation and elongated the life span of the RFS‐TENG.^[^ ^51^, ^52^ ^]^ In this work, 250 *μ*m FEP film and 5 mm gap were chosen for the excellent triboelectrification performance. As shown in Figure 1b, when the FEP sweeps on the nylon, the FEP losses electrons while the nylon gains electrons owing to their electron affinity difference. The positive and negative charges continue accumulating until the triboelectrification saturation after a few seconds. The displacement between the FEP and the nylon results in the voltage drop for charging the LL. There are six pairs of Ag electrodes connected in parallel in the PCB corresponding to six bent FEP films, which means that the output electrical frequency of the RFS‐TENG is six times of that of the mechanical stimuli. As shown in Figure 1b, the charge transfer process from the RFS‐TENG to the LL consists of four stages for one electric cycle of the RFS‐TENG. The left‐sided two insets illustrate the cross‐sectional diagram of the LL with and without tribo‐charging at stages 1 and 4. The LL is charged by the TENG as described above, which alternatively changed twice in one electric cycle. Therefore, the optical oscillation frequency of the developed system is 12 times of the input mechanical frequency, and the majority of the environmental mechanical stimuli are with the frequency of above 2 Hz, making the optical switching frequency of above 24 Hz, which is higher than the vision capability of the human eyes.

**Figure 2:**
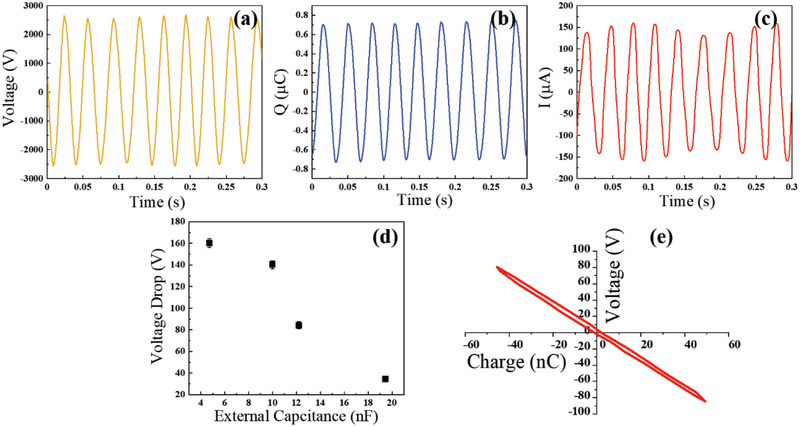
Electrical characterization of the RFS‐TENG and the LL triggered by the RFS‐TENG. a‐c) Open‐circuit (OC) voltage, short‐circuit (SC) charge transfer, and current of the RFS‐TENG operating at 300 rpm rotation speed. d) The relationship between the voltage drop on the LL charged by the RFS‐TENG with the external capacitance. e) The relationship between the voltage drop *V* of the LL and the charge transfer *Q* on the LL when the LL was tribo‐charged by the RFS‐TENG operating at 30 rpm with the external capacitance of 12 200 pF. Each point in (d) was tested for five times. The values of the voltage drop in (d) are presented as mean value plus standard deviation.

Before investigating the optical behavior of the developed self‐powered smart color tuner driven by the RFS‐TENG, the electrical characteristics of the RFS‐TENG and the developed color tuner were first evaluated, through the electrometer Keithley 6514. To characterize the open‐circuit (OC) voltage of the developed RFS‐TENG, a 2000M Ohm resistor was serially connected with the RFS‐TENG and an electrometer as a current meter.^[^ ^53^ ^]^ The RFS‐TENG was driven by a motor with the rotation speed of 300 rpm. The OC voltage was given by the product of the 2000M Ohm resistance and the current measured, as shown in **Figure** **2**a. The peak OC voltage was measured to be above 2500 V. It should be noticed that such the high voltage is not harmful for electronic devices such as LDs due to the low charge output (nC level) of the TENG. In the meanwhile, the TENG is not used to power the LD, due to the huge impedance mismatch between them.

Afterward, the electrometer as the coulomb meter was directly connecting to the RFS‐TENG to measure its SC charge transfer. The mechanical stimulation is identical to that for the OC voltage measurement as mentioned above. As shown in Figure 1b, the maximum charge transfer can be as high as 1400 nC, thanks to the soft‐contact enabled triboelectrification. As calculated from the OC voltage and the maximum charge transfer, the inner capacitance of the RFS‐TENG is about 350 pF. By using the same characterization circuit with the electrometer in the current meter function, the SC current was evaluated as well, as shown in Figure 2c.

To characterize the relationship between the voltage drop on the LL and the external capacitance, various external capacitors were connected in parallel to the LL, and the voltage drop is measured in Figure 2d. The shortest focal length can be obtained when the voltage drop is tuned around 120–150 V. The electrical model of the TENG‐charged LL was investigated. A measurement circuit was constructed to reveal the relationship between the voltage drop *V* of the tribo‐induced LL and the charge transfer *Q* when 12 200 pF external capacitor was connected in parallel, as indicated in Figure S3 in the Supporting Information where two electrometers were employed as the coulomb and volt meters, respectively. The *V–Q* relationship is illustrated in Figure 2d, which is two nearly overlapped straight lines during charging and discharging, indicating that the electrical model of the LL is a pure capacitor, the capacitance of which is almost not affected upon the tribo‐charging. The small area surrounded by the charging and discharging lines may arise from the electrolyte discharging during the fluidic actuation process.^[^ ^44^ ^]^ Considering the 300 pF inner capacitance of the Keithley 6514 electrometer as the voltmeter,^[^ ^53^ ^]^ the capacitance of the LL is calculated to be around 255.6 pF.

**Figure 3:**
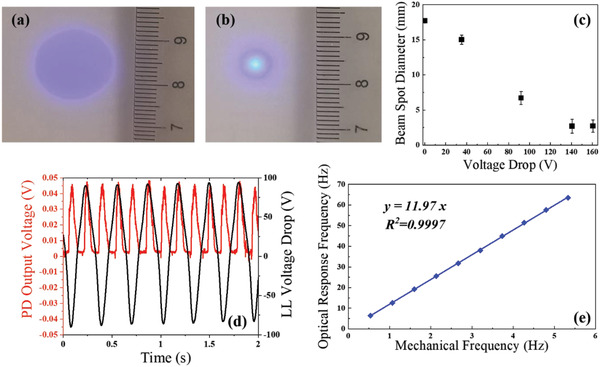
Optical characterization of the LL before and after triggered by the RFS‐TENG. a,b) The blue light beam spot after passing through the LL before and after tribo‐charging, where the distance between the LL and white background is 10 cm, the external capacitance is 4700 pF and the rotation speed of the RFS‐TENG is 300 rpm. c) The relationship between the voltage drop on the LL and the beam spot diameter illuminated on the 10 cm away white background. d) The synchronously measured voltage drop on the LL and the PD output voltage. e) The relationship between the optical response frequency enabled by the tribo‐charged LL and the mechanical frequency. Each point in (c) was tested for five times. The values of the beam spot diameters in (c) are presented as mean value plus standard deviation.

The tribo‐charging induced optical behavior of the LL is characterized. The optical characterization setup is shown in Figure S4 in the Supporting Information. A collimation lens of 8 mm in front of the LD was used to generate the collimated blue light. The collimated blue light passed through the LL concentrically situated 10 mm far away from the collimation lens, which is electrically connected with the RFS‐TENG and a 12 200 pF external capacitor. The rotation speed of the RFS‐TENG is kept at 300 rpm. A white background was set perpendicularly to the incident light blue light, with the distance of 10 cm to the LL. The illuminated blue light beam spot is independent on the injection current of the LD, which is tuned to achieve the best visualization performance. As shown in **Figure** **3**a,b, when the LL is not charged, the blue light intensity is uniformly distributed on a circle with a diameter of 16 mm. And after the LL was tribo‐charged, the beam spot presented the highest intensity in its central area, with the diameter of around 2 mm. By changing the external capacitance to tune the voltage drop on the LL, the beam spot diameters under different voltage drops were characterized, as indicated in Figure 3c. It can be observed that with the voltage drop increasing from 0 to 140 V, the beam spot decreases with the nearly linear relationship between the spot diameter and the voltage drop, attributed to the tribo‐charging induced electrowetting effect. When the voltage drop is further increased to 160 V, the beam spot diameter remains unchanged, achieving the saturated electrowetting. The stimuli frequency of the TENG is set at 5 Hz (300 rpm), making the optical switching frequency of 60 Hz, beyond the vision capacity of the human eyes. Video S1 in the Supporting Information shows the illuminated beam spot during the tribo‐charging, playing in the normal and 50‐time slower speeds, respectively.

Through the tribo‐charged LL, the environmental mechanical motion captured by the TENG can be reflected by the change of the beam spot size, which can be visualized through the constructed two‐channel, optoelectrical synchronous measurement system as shown in Figure S5 in the Supporting Information. The optical intensity can be detected by a photodetector (PD) by the output voltage in the linear relationship. The active area in the PD is around 4 mm^2^, which is located in the central position on the beam spot with tribo‐charging. The Keithley 6514 electrometer as the voltmeter was connected to the LL with a 12 200 pF external capacitor in parallel. When the RFS‐TENG was triggered, the measured PD output voltage peak is 0.45 V, shown in Figure 3d. It is observed that the peak PD output voltages achieved at both the positive and negative peak LL voltage drops, regardless of the current and voltage directions. As a result, the optical switching frequency is twice of that of the TENG output. The linear relationship between the optical response frequency measured by the PD and the input mechanical frequency of 0.5–5.5 Hz is revealed in Figure 3e, with a fitted slope of 11.97, as consistent with the expected ratio of 12, indicating the self‐powered wireless sensing capability through the tribo‐induced mechanical‐electrical‐optical signal conversion.

**Figure 4:**
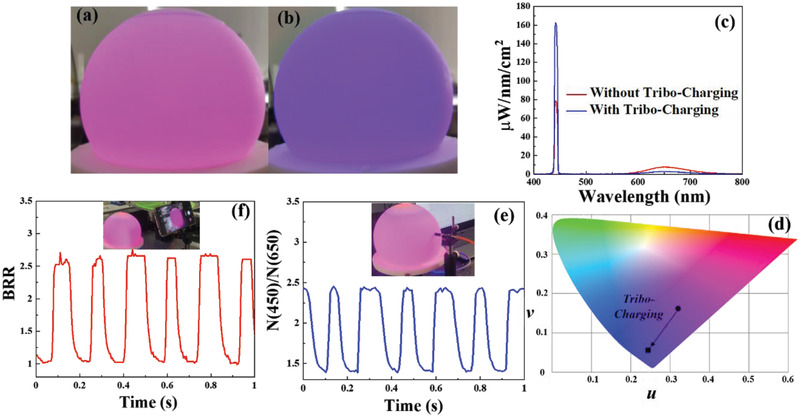
Optical characterization of the developed tribo‐induced color tunable SSL system. a,b) The photographs of the illuminated color‐tunable SSL system before and after tribo‐charging. c,d) The SPD and color coordinate in CIE1960 color space comparison of the developed color‐tunable SSL system with and without tribo‐charging. e) The measured time‐variant CCD‐captured light intensity ratio at the wavelength between 450 and 650 nm, where the Maya2000Pro spectrophotometer set at the high‐speed acquisition mode was employed. The integration time during the acquisition process is 7 ms for each spectrum. The inset in (e) shows the spectral characterization setup, where a fiber connected with a cosine corrector was employed for collecting the diffusive light. f) The camera‐characterized time‐variant intensity ratio between blue and red channels ranging from 0 to 255, which can be processed by the developed computer program. The camera employed was Huawei Mate20 smartphone camera, where the frame rate was set at 240 fps. The camera monitoring setup is shown in the inset of (f). For (a)–(d), the external capacitance was 4700 pF. For (e) and (f), the external capacitance was 12 200 pF. The RFS‐TENG was operated at 300 rpm rotation speed.

To clearly visualize the variation of the beam spot size by regular cameras, we introduced the color‐tuning capability in the tribo‐charged LL assisted SSL system, which is enabled by the fluorescent and scattering materials as structured in Figure 1. The CCP and a 3D printed diffuser were integrated with the tribo‐charged LL assisted SSL system to achieve the color‐tuning function. The operating current is chosen at 300 mA, corresponding to the 3.3 V voltage drop. Since the color‐tuning capability is the main focus of this work rather than the power and efficiency investigation, a relatively low injection current was selected to avoid the potential issues brought by the phosphor self‐heating and the thermal accumulation inside the P–N junction of the LD.^[^ ^54^, ^55^, ^56^ ^]^ The illuminations are compared before and after the tribo‐charging, with the photographs shown in **Figure** **4**a,b. To achieve the highest color variation, a 4700 pF external capacitor was connected and the RFS‐TENG rotation speed was set at 300 rpm. It can be clearly observed that after the tribo‐charging, the SSL system changed its color from pink to purple. Besides, the illumination is spatially uniform, which is attributed to the strong scattering capacities of TiO_2_, the isotropic emission property of the re‐emitted fluorescent light and the light randomization capability enabled by the 3D printed diffuser. Video S2 (Supporting Information) shows the illumination color variation of the color‐tunable SSL system upon the RFS‐TENG was triggered. It can be observed that the color response upon tribo‐charging is very fast and thus the fluidic actuation delay cannot be identified.

A spectrophotometer, Maya2000Pro, was employed to measure the spectral power distribution (SPD) and the color characteristics of the developed color‐tunable SSL system before and after the tribo‐charging. The constructed spectral characterization setup is shown in Figure S6 in the Supporting Information, where the optical fiber was fixed with a cosine corrector attached to the SSL system for diffusively sampling the incident illumination light. During the measurement, the integrating time was set to be 500 ms, much longer than the period of the color‐oscillation 16 ms, meaning that the measured SPD is the integral average of a host of SPDs during the optical integration process. Before undertaking the measurements, the spectrophotometer was calibrated by a standard lamp, HL‐3P‐CAL produced by Ocean Insight Ltd, considering the sensitivity dependence on the charge‐coupled device (CCD) inside the spectrophotometer on the wavelength ranging from 300 to 1100 nm. The measured SPDs under the two states are compared as shown in Figure 4c. From Figure 4c, it can be identified that the output spectrum consists of two parts: narrow‐band LD emitted blue light spectrum and broadband fluorescent emission spectrum. And when the tribo‐charging was performed, the blue light spectral power was nearly doubled, and the spectral power of the fluorescent light with charging became one‐quarter of that without charging. Under tribo‐charging, even though the area of the TiO_2_ layer covers the whole beam spot, the fluorescent light emission arose from the interaction between the sideward‐scattered blue light and the phosphor. The luminous value of the SSL system without and with tribo‐charging are measured to be 78 and 46 lm, respectively. The decrease of the luminous value with tribo‐charging is due to the blueshift of the illumination wavelength. To intuitively visualize the extent of the color shift, CIE1960 color space was employed and the measured SPD was calculated into the color coordinate (*u*, *v*).^[^ ^57^, ^58^ ^]^ As shown in Figure 4d, the color coordinates (*u*, *v*) without and with the tribo‐charging were plotted in the CIE1960 color space, and the color‐shift distance of 0.1114 was observed. Considering the below −0.001 color coordinate uncertainty as the noise level of the majority of SSL system, the SNR of the developed paradigm is above 111.45, which is high enough for wireless sensing.

To evaluate the time‐variant color performance for the wireless sensing purpose, the spectrophotometer was set with the high‐speed acquisition mode, where the measurement setup remained unchanged as indicated in the inset of Figure 4e. To proceed with the continuous high‐speed spectral intensity sampling, the CCD captured photon number *N*(*λ*) at the wavelength *λ* rather than the SPD after the calibration over a given time interval was sampled. At the high‐speed mode, the integration time of the spectrophotometer was set to be 7.2 ms, giving a sampling rate of 138.89 Hz. If we define that ten‐time sampling in a period can fully reveal a mechanical motion profile, the effective range for the rotation sensing based on the developed paradigm is from 0 to 166.67 rpm. To minimize the sensing inaccuracy owing to the fluidic actuation delay of the LL, ^[^ ^44^, ^59^, ^60^ ^]^ a lower voltage drop should be loaded on the LL and the external capacitance changed its value from 4700 pF to 12 200 pF. Taking the sensing under the 30 rpm rotation speed as an example, the *N*(*λ*) evolvement process in one period is illustrated in Figure S7 in the Supporting Information. Since the blue light and fluorescent light emission peak at 450 and 650 nm, respectively, the *N*(*λ*) variation over the full spectrum can be simply represented by *N*(450)*/N*(650). The time‐variant *N*(450)/*N*(650) under the RFS‐TENG rotation speed at 30 rpm is illustrated in Figure 4e. It can be identified that the time‐variant *N*(450)/*N*(650) enables the motion profile to be clearly revealed with a 12‐time enhanced frequency. The bandwidth difference between two adjacent peaks may arise from the fact that the LL takes the time to recover its shape when the electric field direction is changed.

The high‐frequency color‐tuning‐based sensing can be captured by cameras, for example, everyone‐owned smartphone, with usually high frame rate for imaging. Taking the Huawei Mate20 and Samsung Galaxy S20 for instances, the highest frame rate can be as high as 980 frames per second (fps). Besides, the zoom in and out functions of the camera enable the sensing to be wirelessly achieved at a long distance. Beside smartphones, CCTVs are also everywhere in both indoor and outdoor environments. In this work, the Huawei Mate20 was employed to measure the time‐variant color, and the frame rate was chosen to be 240 fps. A Python program was written for the image processing, where the OpenCV library was employed. In the developed paradigm, the behavior variation of the majority of incident photons occurred at B (blue) and R (red) channels. Therefore, B to R ratio (BRR) was defined to characterize the color variation performance monitored by the camera. The time‐variant BRR was characterized with the RFS‐TENG rotation speed of 30 rpm, as shown in Figure 4f. The camera‐based monitoring system is shown in the inset of Figure 4f. It can be observed that the camera monitored profile is nearly identical to that characterized by the spectrophotometer shown in Figure 4e, demonstrating the effectiveness of the self‐powered wireless sensing using the camera as the signal receiver. Video S3 illustrates the color variation of the SSL system in a few seconds, as played in the 20‐time slower speed.

**Figure 5:**
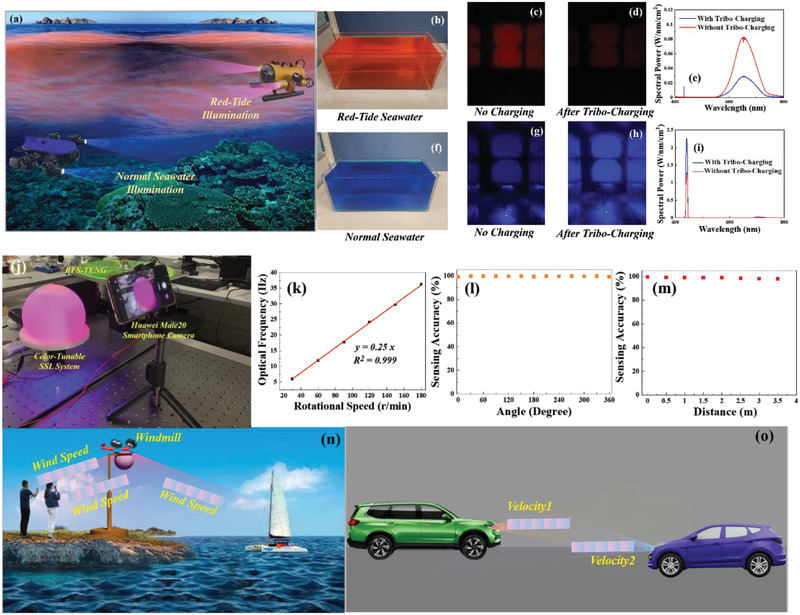
Underwater photographing and wireless sensing applications of the developed tribo‐induced color tunable SSL system. a) Schematic diagram of the color‐tunable underwater illumination system using the developed paradigm which can be carried by the AUV for the wide‐range underwater detection purpose. b,f) Photographs of the simulated red‐tide and normal seawaters, where the water in the tank was dyed by the food colorings. c,d) The photograph imaged by the camera through the simulated red‐tide seawater without and with tribo‐charging. g,h) The photograph imaged by the camera through the simulated normal seawater without and with tribo‐charging. e,i) Characterization of the illumination light passing through the simulated red‐tide and normal seawaters with and without tribo‐charging by the spectrophotometer. j) Constructed setup to achieve the wireless sensing of the rotation speed. k) The relationship between the optical frequency and the rotation speed. l–m) The dependence of the sensing accuracy on the sensing angle and sensing distance. n) Schematic diagram of the self‐powered wind‐speed wireless sensing employing the developed tribo‐induced color tuner, where the wind speed information can be wirelessly received by the camera through the time‐variant color. o) Schematic diagram of the self‐powered wireless vehicle‐to‐vehicle communication system for exchanging the vehicle speeds of two vehicles. For a–i), the external capacitance is 4700 pF with the RFS‐TENG rotation speed of 300 rpm. For k–m), the external capacitance is 12 200 pF while the rotation speed varies.

The developed color‐tunable SSL system benefits both color‐tunable smart lighting and self‐powered wireless sensing. The color‐tunable environmental lighting system is important to achieve the optimal illumination quality, since the environmental absorption and scattering properties vary with the time and spatial distributions, corresponding to the different optimal spectra for the highest lighting quality. Taking seawaters for instances, the spectral attenuation property of the seawater is affected by several factors including sunlight irradiation, distributions of zooplankton and phytoplankton, and seawater pollutions, etc.^[^ ^61^ ^]^ Thus, the preferred illumination spectrum for underwater lighting varies with the seawater depth, time and spatial distributions. Different with the majority of blue seawater where the blue light presents the longest penetration distance, the red‐tide seawater presents a red appearance which absorbs a broad range of light except the red one. However, until now, the conventional SSL systems for wide‐range underwater detection fail to achieve effective illumination when the seawater condition is changed. To achieve this goal, the developed color‐tunable SSL system was employed to achieve the smart underwater lighting, which can be easily integrated with the autonomous underwater vehicles (AUV) where the RFS‐TENG can be driven by the water‐flowing energy, with the schematic diagram of the demonstration illustrated in **Figure** **5**a. In our demonstration, an acrylic‐based water tank with the dimension of 45 cm × 20 cm × 20 cm was prototyped and filled with the water. The water was blue and red dyed by the food colorings to simulate the normal and red‐tide seawaters, respectively, as shown in Figure 5b,f. The emitted light of the developed SSL system after passing through the 45 cm long simulated seawater was imaged by the camera and sampled by the spectrophotometer via a fiber, the setup of which is shown in Figure S8 in the Supporting Information. The characterization with and without tribo‐charging were performed for both types of simulated seawaters, and results from the camera and the spectrophotometer are shown in Figure 5c–e,g–i. It can be identified that the non‐charged SSL is preferred for the red‐tide seawater, where a bright red contour of the SSL system was clearly imaged, and the SPD ranging from 550 to 750 nm is around three times of that of tribo‐charged SSL. In contrast, as for the normal seawater as indicated in Figure 5g–i, the tribo‐charged SSL system with the higher blue light emission component is preferred to achieve the best illumination. Therefore, the developed color‐tunable SSL system is demonstrated to be effective to achieve high‐quality underwater photographing.

The developed self‐powered color tuner can also be employed for achieving the battery‐free wireless sensing of the rotation speed, with the camera serving as the signal receiver. For examples, the developed TENG can be integrated with a windmill to wirelessly monitor the wind speed. Besides, the TENG can be connected with a turbine where the water flow can trigger the rotation of the TENGs,^[^ ^23^ ^]^ enabling the wireless sensing of the water flow. The optical frequency measured by the fast‐sampling spectrophotometer or the camera can reveal the rotation speed of the TENG as discussed above. To evaluate the capability and accuracy of the developed paradigm, the wireless sensing was performed to detect the rotation speeds using the setup as shown in Figure 5j, and the results are shown in Figure 5k–m. As shown in Figure 5k, excellent linearity was observed between the optical response frequency and the rotation speed ranging from 0 to 180 rpm, with a high sensing accuracy. The sensing range is limited by the frame rate of the camera, which is 180 rpm (corresponding to 36 Hz in optical response frequency) in this experiment. Thanks to our 3D printed diffuser, the time‐variant color illumination is spatially uniform as indicated in Figure 5l, independent of the angle. The sensing was also performed at different distances up to 3.5 m. As shown in Figure 5m, the sensing accuracy is not affected by the distance. With the assistance of the zoom‐in capability of the smartphone camera, the maximal effective sensing distance should be as high as 500 m. Therefore, as a self‐powered wireless sensor, the developed system was demonstrated for speed monitoring.

As shown in Figure 5n, the developed paradigm can be employed to achieve the wireless sensing of the wind speed, where the RFS‐TENG is driven by a windmill. The windmill rotation frequency reflecting the wind speed can be monitored by the camera. This technology is particularly significant to the coastal area, where the airflow information plays an important role in affecting the human activity and the operation of ships. Besides, as illustrated in Figure 5o, the developed paradigm benefits the vehicle‐to‐vehicle communication system, where the vehicle speed information can be detected through the rotation speed and exchanged between two vehicles to minimize the collision hazard at complex traffic conditions. The vehicle velocity of the coming, leading or following car can be monitored in an in‐situ way by the camera‐based devices, such as the traveling data recorder (TDR), etc.

## 3 Discussion

In this work, a tribo‐induced color tuner was developed through the combination of an RFS‐TENG, a LL and a CCP, for applications in smart lighting and self‐powered wireless sensing. The developed color tuner can be easily incorporated into a commercial SSL system, where the color‐tuning capability was achieved by tribo‐charging without consuming additional electrical energy. Through the tribo‐charging, the SSL system changed its illumination color from pink to purple, presenting a wide‐range color‐shift distance of 0.1114 in the CIE1960 color space. Through the color‐tuning scheme, a smart underwater photographing system was performed, which can achieve excellent imaging quality at both normal and red‐tide seawater conditions. The developed color tuner is also a paradigm‐shift solution for self‐powered wireless sensing. Unlike conventional active sensors which suffers from involvements of bulky and expensive electrometers, cable connections, and high‐power consumption, the developed sensing system is in wireless form without additional energy consumption, and the signal can be sent by everywhere‐existed lamps and processed by everyone‐owned cameras or CCTVs. The color‐tuning‐based wireless sensing also presents a high SNR, which is estimated to be above 111.45. Employing the developed system and a commercial smartphone camera, the self‐powered wireless sensing of the rotation speed was performed. The sensing accuracy can be as high as above 98% within the range of 0–180 rpm independent of direction and distance. A broad application using the developed paradigm can be anticipated ranging from human‐centric or environment‐centric smart lighting systems to achieve the comfortable visualization experience or high‐quality photographing, and the self‐powered wireless sensing system for monitoring air flows, ocean currents, and vehicle speeds, etc. With the rapid development of CCTVs and great penetration of SSL system for general lighting, the developed paradigm has the great potential to achieve the wireless sensing network without the needs of batteries, signal amplification, and cables.

## 4 Experimental Section

##### Fabrication of RFS‐TENG

The developed RFS‐TENG consists of a stator and a rotator. To fabricate the stator, the PCB technology was employed where Ag was deposited on the flame retardant 4 (FR4) substrate, serving as the electrodes. The Ag on the rectangle‐shaped FR4 was patterned into two parts: six electrically connected, uniformly distributed sectors and the remaining area. The two parts were segmented with the gap of 6 mm. A 50 *μ*m thick nylon film was taped above the Ag layer, serving as the positive triboelectric material. To fabricate the rotator, a 3 mm thick acrylic was laser‐machined to be a 15 cm diameter circle embedded with six uniformly distributed 105 mm × 2 mm holes. Six sector‐shaped 250 *μ*m thick FEP films were inserted into these holes and fixed by the tapes, serving as the negative triboelectric material.

##### Fabrication of the CCP

A circled substrate with two 2 mm deep concentric cavities was first 3D printed using the diffusive resin material. The two cavities were to be filled by (Sr, Ca)AlSiN3:Eu and TiO_2_ encapsulants, the diameters of which were 21 and 4.8 mm, respectively. Before dispensing, both particles were mixed with the transparent resins, the weight mixing ratios of which are 0.3:1 ((Sr, Ca)AlSiN3:Eu:resin) and 0.05:1 (TiO_2_:resin). After blending and vacuuming, the mixtures were dispensed onto the two cavities. The CCP was ready for use after 2 h curing for the solidification.

##### Assembling the Self‐Powered Color Tuner into the SSL System

A metal‐based fixture was machined for the purpose of the LD heat dissipation and concentric connection between the LD and the collimation lens. To concentrically connect the tribo‐induced LL and the LD fixture, an L‐shaped mechanical connector was 3D printed, where the LD fixture and the LL were connected with the two sides of the L‐shaped connector through the nylon screws. Afterward, a 3D printed diffuser integrated with the CCP was connected with the L‐shaped connector via screwing.

##### Synchronous Optoelectrical Characterization of Tribo‐Induced LL

The LL connecting with the RFS‐TENG was integrated with the LD using the L‐shaped connector, as described above. The PD was concentrically located 10 cm far away from the LL. A Keithley 6514 electrometer functioning as the voltage meter was connected with the LL. When the LL was powered by the RFS‐TENG, the voltage drop on the tribo‐charged LL and the intensity of the LD emitted light after transmitting through the tribo‐charged LL were measured in the meanwhile, the data of which was sampled by the two‐channel NI‐cDAQ synchronous data acquisition system.

##### Statistical Analysis

The measured time‐variant transferred charges, SC currents and OC voltages of the TENG, time‐variant PD output voltages, SPDs and time‐variant spectra were the original data measured by the Keithley6514 electrometer, PD and spectrophotometers, which do not need the preprocessing process. The optical frequency is obtained through the fast Fourier transformation (FFT). The FFT operation was finished by the OriginPro software. The time‐variant BRR was obtained by the ratio of the intensity in the B channel to that in the R channel, where the time interval is determined by the reciprocal of the frame rate of the camera. The processing of the time‐variant BRR is finished by the developed Python program. The beam spot diameters of the blue light and the voltage drops on the LL upon loading the external capacitor were presented in form of mean plus standard deviation, resulting from five independent experiments for each point. This statistical analysis is finished by the OriginPro software.

## Conflict of Interest

The authors declare no conflict of interest.

## Author Contributions

J.W. and H.W. contributed equally to this work. J.W., H.W., K.Y., and Y.Z. conceived the idea, discussed the results, and prepared the manuscript. J.W. designed and fabricated the CCP, RFS‐TENG, and prototyped the color‐tunable SSL system. J.W. and H.W. designed the whole experiments and constructed the measurement setups. J.W. undertook the optoelectrical characterization of RFS‐TENG, the tribo‐induced LL, and the color‐tunable SSL system and analyzed the characterized data. K.Y. offered his expertise on the implication of marine applications. Y.Z. helped on discussions and preparation of the manuscript.

## Supporting information

Supporting Information

Supplemental Video 1

Supplemental Video 2

Supplemental Video 3

## Data Availability

Research data are not shared.
