# Supplementary material for: Tribo‐Induced Color Tuner toward Smart Lighting and Self‐Powered Wireless Sensing
Source: Adv Sci (Weinh). 2021 May 2;8(12):2004970. doi: 10.1002/advs.202004970 (PMC8224429; doi:10.1002/advs.202004970)
Supplement: Supplementary file 1 — Supporting Information [file ADVS-8-2004970-s004.pdf]

© 2021 Wiley-VCH GmbH

ADVANCED SCIENCE
Open Access

Supporting Information

for *Adv. Sci.,* DOI: 10.1002/advs.202004970

Tribo-Induced Color Tuner for Smart Lighting and Wireless Sensing Applications

Jiaqi Wang[1, 2], *Haoyu Wang*[2], *Kedong Yin*[3], *Yunlong Zi*[2, *]

Supplementary Information

Tribo-Induced Color Tuner for Smart Lighting and Wireless Sensing Applications

Jiaqi Wang[1, 2], Haoyu Wang[2], Kedong Yin[3], Yunlong Zi[2, *]

[1.] School of Marine Sciences, Sun Yat-Sen University, Zhuhai 519082, China.

[2.] Department of Mechanical and Automation Engineering, The Chinese University of Hong Kong, Shatin, N.T., Hong Kong, China.

[3]. Southern Marine Science and Engineering Guangdong Laboratory (Zhuhai), Zhuhai 519080, China.

*Corresponding author, email: ylzi@cuhk.edu.hk

1. Operation mechanism of the liquid lens.

As shown in Fig. S1, the operation mechanism of the employed liquid lens is illustrated. As indicated in Fig. S1 (a), the dielectric oil presents a concave-lens performance at the normal state without being charged, diverging the incident light. When the LL is charged, as shown in Fig. S1 (b), the dielectric oil is convex in shape, focusing the light.

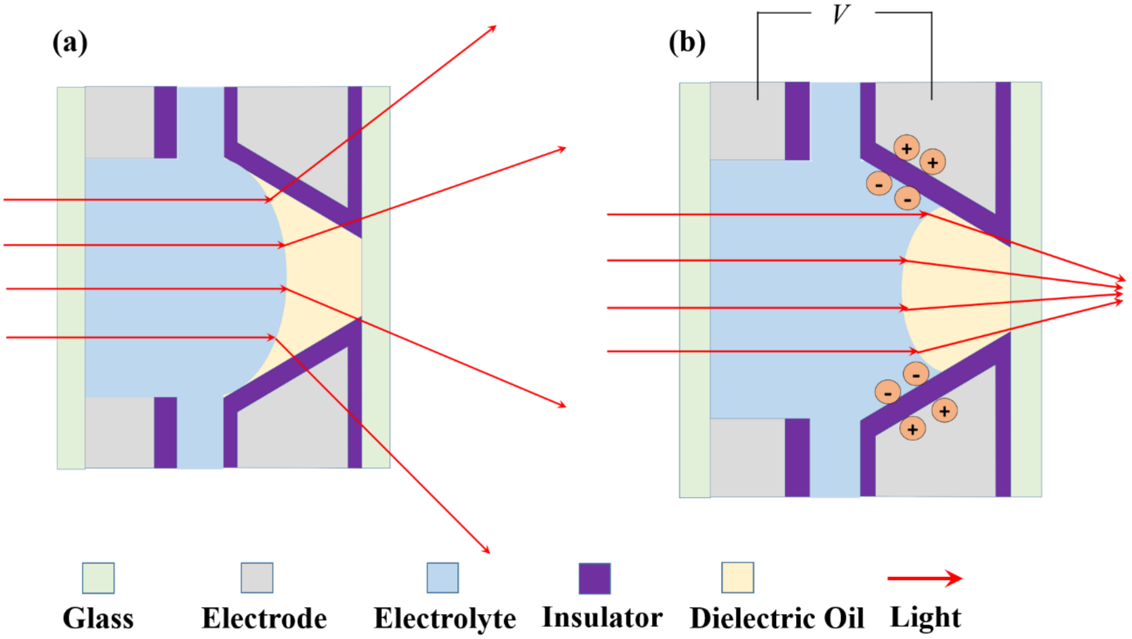

Figure S1. Schematic diagram of the operation mechanism of the LL. a) Without charging. b) With Charging.

2. Scattering pattern characterization of (Sr,Ca)AlSiN$_3$:Eu and TiO$_2$ particles using the Lorenz-Mie theory.

The angular scattering pattern of a particle reveals the angular intensity distribution of the scattered light after being scattered. To obtain the angular scattering pattern, the Lorenz-Mie theoretical calculation were performed for both (Sr,Ca)AlSiN$_3$:Eu and TiO$_2$ particles. The Lorenz-Mie theory was derived by solving the Maxwell equation under the spherical boundary condition and provided an analytical solution to the scattering field of a sphere.

The input parameters including refractive indices (RIs) of the spherical particle and the medium and the particle radius are necessary to perform the calculation. The extension cross section is the sum of the absorption and scattering cross sections, which in Lorenz-Mie theory can be represented by

$$C_{sca} = \frac{2\pi}{k^2}\sum_{0}^{\infty}(2n+1)(|a_n|^2+|b_n|^2) \tag{S-1}$$

$$C_{ext} = \frac{2\pi}{k^2}\sum_{1}^{\infty}(2n+1)\,\mathrm{Re}(a_n+b_n) \tag{S-2}$$

$$C_{abs} = C_{ext} - C_{sca} \tag{S-3}$$

where C_{ext}, C_{Sca}, C_{Abs} are the extinction, scattering and absorption cross sections, $k = 2\pi/\lambda$ is the wave number, and a_n and b_n are the expansion coefficients with even symmetry and odd symmetry, respectively. As shown in Eq.(S-4) to Eq.(S-5), a_n and b_n are derived as

$$a_n = \frac{m\psi_n(mx)\psi_n'(x) - \psi_n'(mx)\psi_n(x)}{m\psi_n(mx)\xi_n'(x) - \psi_n'(mx)\xi_n(x)} \tag{S-4}$$

$$b_n = \frac{\psi_n(mx)\psi_n'(x) - m\psi_n'(mx)\psi_n(x)}{\psi_n(mx)\xi_n'(x) - m\psi_n'(mx)\xi_n(x)} \tag{S-5}$$

where x is the size parameter ($x = kr$, r is the radius of the sphere), m is the relative refractive index of particles, and $\xi(x)$ and $\psi(x)$ are Riccati-Bessel functions.

The analytical solution in the spherical coordinate gives the functions in series. The angular scattering profile can also be calculated by the Lorenz-Mie theory, which can be represented by

$$S_1(\theta) = \sum_{n=1}^{\infty}\frac{2n+1}{n(n+1)}\left[a_n\frac{P_n^1(\cos\theta)}{\sin\theta} + b_n\frac{dP_n^1(\cos\theta)}{d\theta}\right] \tag{S-6}$$

$$S_2(\theta) = \sum_{n=1}^{\infty} \frac{2n+1}{n(n+1)} [b_n \frac{P_n^1(\cos\theta)}{\sin\theta} + a_n \frac{dP_n^1(\cos\theta)}{d\theta}] \quad \text{(S-7)}$$

$$\beta(\theta) = \frac{1}{2}[|S_1(\theta)|^2 + |S_2(\theta)|^2] \quad \text{(S-8)}$$

where $S_1(\theta)$ and $S_2(\theta)$ are intensities with perpendicular and parallel polarizations. $P^1{}_n(\cos\theta)$ is the associated Legendre polynomial. $\beta(\theta)$ represents the intensity for intensity with non-polarization. Table. S1 illustrates the RIs and particle radiuses of the materials to undertake the Lorenz-Mie calculations. The calculated results are shown in Fig. S2. It can be identified that for the micro-sized (Sr,Ca)AlSiN3:Eu, a great proportion of the light is forward-scattered, and the sideward-scattering is very trivial. TiO_2, in contrast, performs a strong sideward-scattering capability. The strong scattering capability of TiO_2 guarantees the uniformity of the scattered blue light after being scattering by the high mixing-ratio TiO_2 encapsulation centrally located in the color-conversion plate (CCP).

Table. S1 Refractive indexes and particle radius of the materials for the calculations

Phosphor type	Refractive Index	Particle Radius (μm)
$(Sr,Ca)AlSiN_3:Eu$	1.91	7.5
TiO_2	2.87	0.05
Transparent Resin	1.5	N/A

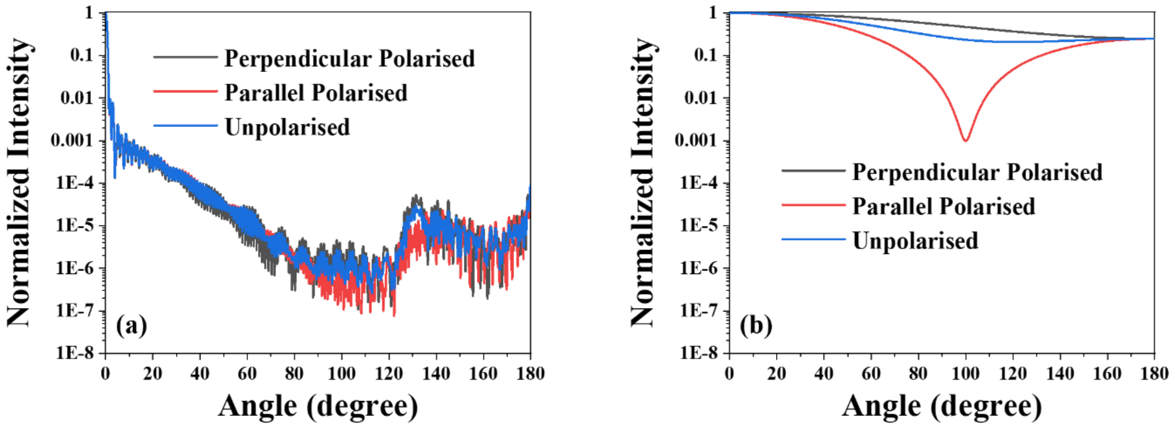

Figure S2. Theoretically calculated angular scattering pattern using the Lorenz-Mie theory. a)

$(Sr,Ca)AlSiN_3$:Eu. b) TiO_2.

3. Electrical characterization of the tribo-charged LL.

To evaluate the relationship between the transferred charge amount Q and voltage drop V of the tribo-charged LL, a synchronous electrical characterization system was constructed, the circuit of which is illustrated in Fig. S3. The equivalent circuit model of the RFS-TENG is the serial connection of an idea voltage source and a time-variant capacitor. The equivalent circuit model of the LL is a capacitance as discussed in the manuscript. Two keithley 6514 electrometers were employed in this measurement circuit, functioning as the voltmeter and coulometer, respectively. A computer-controlled NI-cDAQ synchronous data acquisition system sampled the output signal from two electrometers in a fast speed, the sampling rate of which is 1000 Hz.

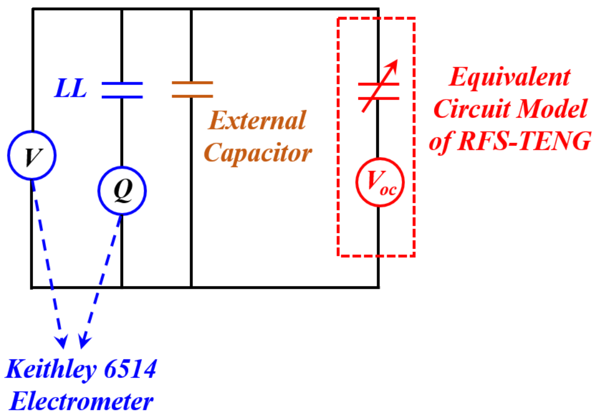

Figure S3. Measurement electrical circuit of the tribo-charged LL to reveal the relationship between the transferred charge amount Q and the voltage drop V.

4. Beam spot diameter characterization of the LD-emitted blue light passing though the tribo-induced liquid lens (LL).

As shown in Fig. S4, a measurement setup was constructed to evaluate the beam spot of the LD-emitted blue light illuminated on the white background 10 cm far away from the LL, after passing through the LL. The beam spots were measured before and after the RFS-TENG was triggered.

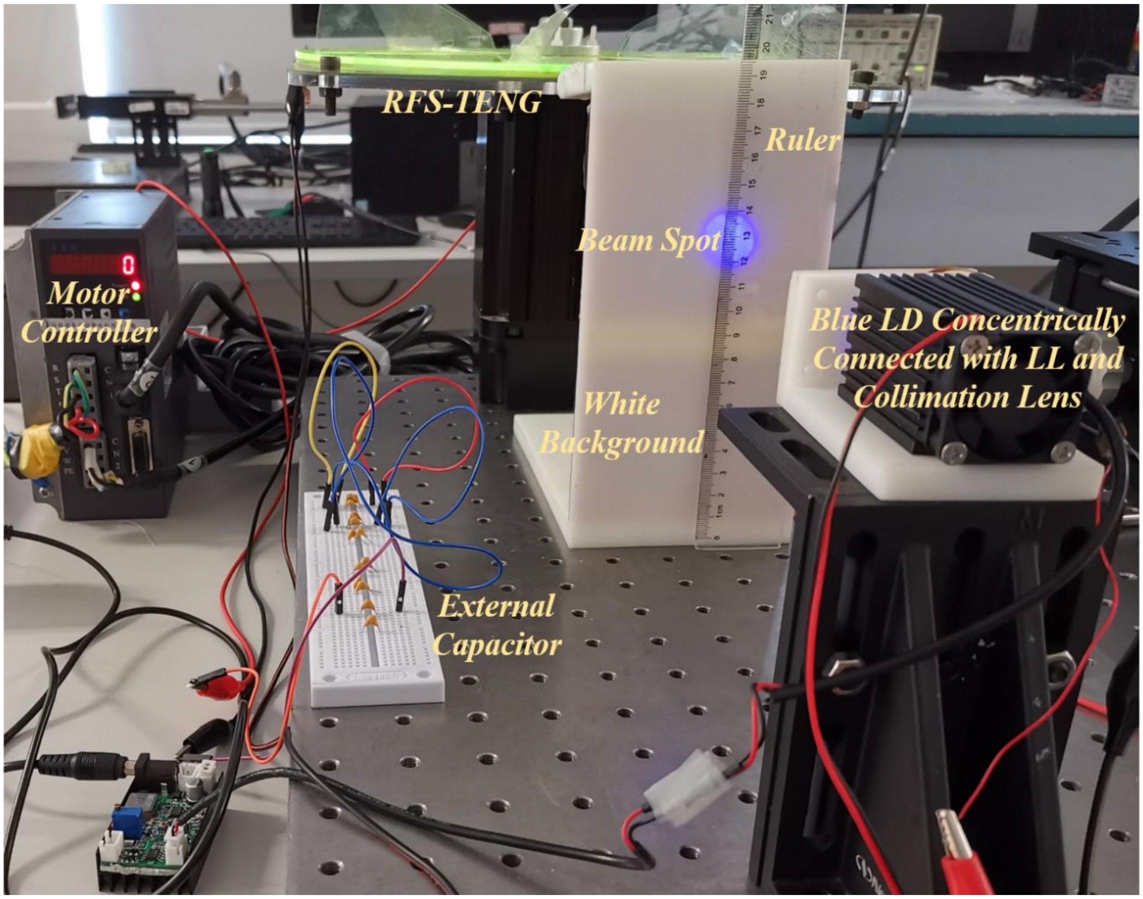

Figure S4. Beam spot diameter characterization setup of the LD-emitted blue light passing though the tribo-induced LL, where the rotation speed of the RFS-TENG is 300 rpm and the external capacitance is 4700 pF.

5. Two-channel opto-electrical synchronous measurement setup to reveal the relationship between the voltage drop and the focusing behavior of the tribo-induced LL.

The white background in Fig. S4 was replaced by a photodetector (PD), where the active area of the PD was positioned at the focal point of the light passing through the tribo-charged LL, as shown in Fig. S5. A Keithley 6514 electrometer functioning as the voltmeter was connected to the LL in parallel to measure its voltage drop. The voltage drop and the PD output voltage were sampled in the meanwhile with the assistance of the NI-cDAQ acquisition system and the LabVIEW software.

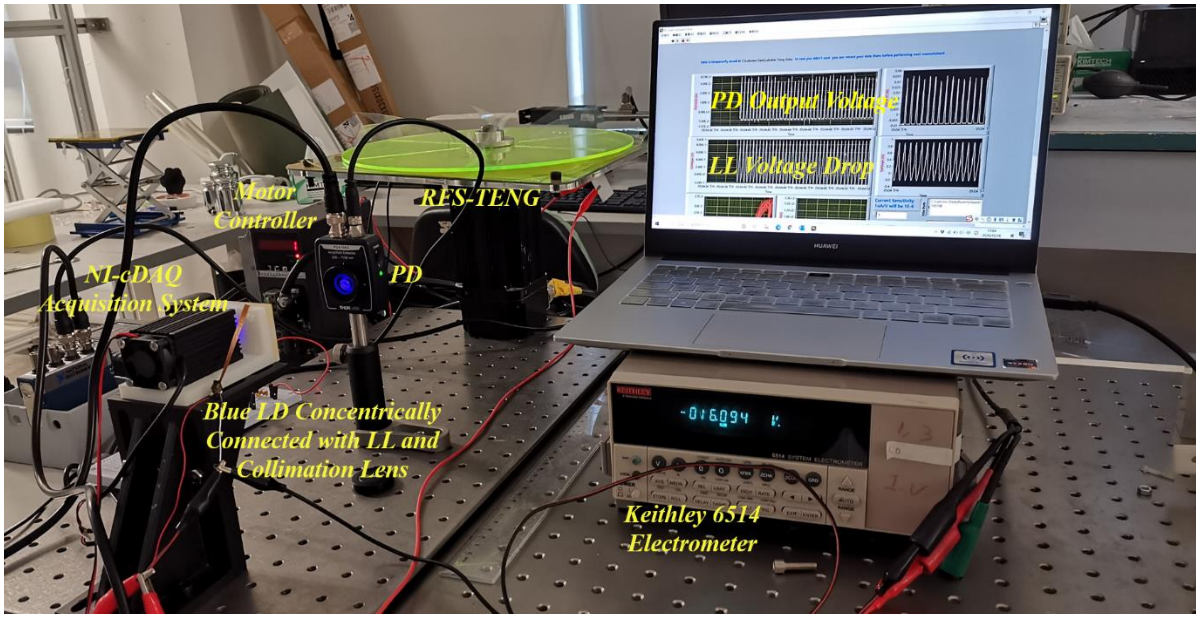

Figure S5. Two-channel opto-electrical synchronous measurement setup to sample the voltage drop on the tribo-induced LL and the PD-detected light intensity of the blue light after passing through the tribo-induced LL in the meanwhile.

6. Spectral characterization of the developed tribo-induced color-tunable solid state lighting (SSL) system.

A commercial spectrophotometer, Maya2000Pro, produced by OceanInsight™, was employed to undertake the spectral characterization of the developed tribo-induced color-tunable SSL system. As shown in Fig. S6, the SSL emitted light was sampled by a fiber of the spectrophotometer. Between the SSL device and the fiber was a cosine-corrector screwed with the the SMA-905 connector of the fiber. The cosine-corrector was able to collect the diffused incident light within a broad incident angle ranging from 0 to 180 degree.

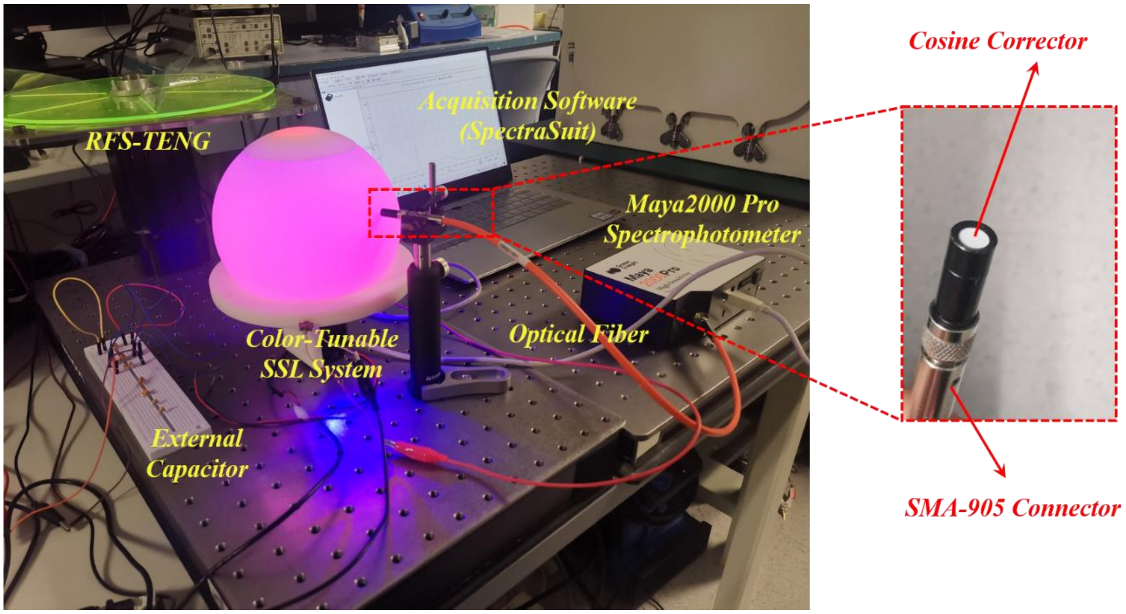

Figure S6. Constructed spectral characterization setup of the tribo-induced color-tunable SSL system based on the Maya2000Pro spectrophotometer.

7. Spectral evolvement in a half period during the color oscillation process.

The high-speed acquisition mode was set for the Maya2000Pro spectrophotometer to evaluate the time-variant color performance. The measurement setup remained unchanged, as shown in Fig. S5. The integration time is 7 ms. The spectral evolvement process in a half cycle when the RFS-TENG operated at 30 rpm is illustrated in Fig. S7.

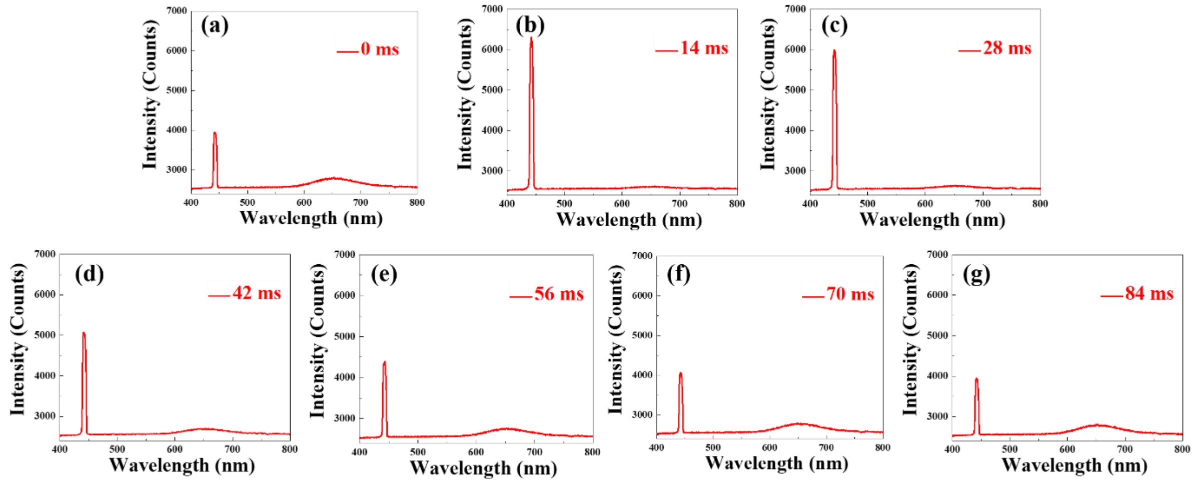

Figure S7. The spectral variation within a half cycle of the developed color-tunable SSL system during the color oscillation process. The rotation speed of the RFS-TENG is 30 rpm and the integrating time of the Maya2000Pro spectrophotometer in the high-speed acquisition mode is 7 ms. From (a) to (g), the integration finishing times are 0 ms, 14 ms, 28 ms, 42 ms, 56 ms, 70 ms and 84 ms, respectively.

8. Image quality evaluation and spectral analysis of the developed color-tunable lighting system under the conditions of the simulated seawaters.

As shown in Fig. S8, a characterization setup was constructed to evaluate the image quality and undertake the spectral analysis of the illumination passing through the simulated seawater using the developed color-tunable SSL system. The light emitted by the SSL system passed through the simulated seawater with the thickness of 45 cm and was finally collected by the smartphone camera and the spectrophotometer, respectively.

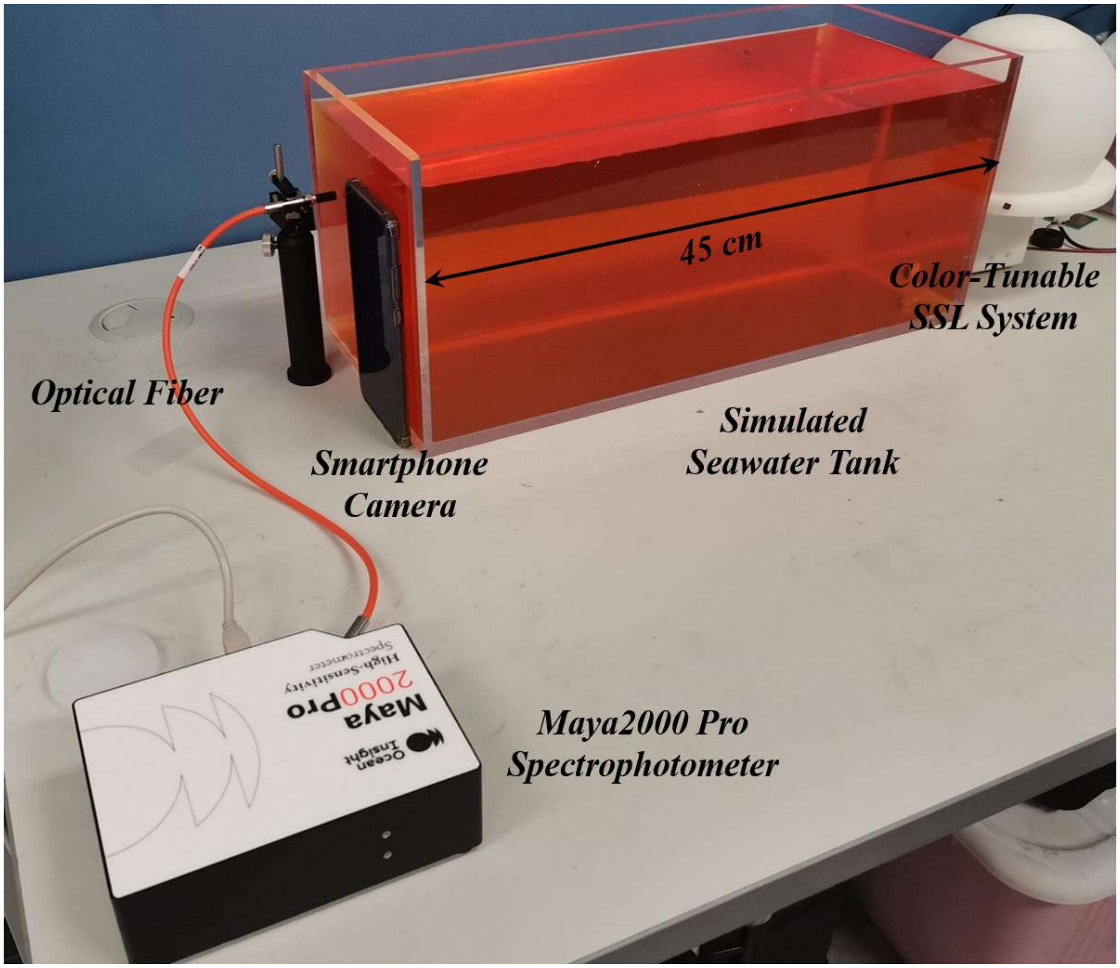

Figure S8. Image and spectral characterization setup of the developed photographing system through the simulated seawater.

Supporting Videos

Video. S1. The blue light beam spot focused by the tribo-induced LL. (The video is played in normal speed (recorded by 30 fps camera) and 50 times slower speed (recorded by 240 fps camera), the camera employed is Huawei Mate20 smartphone camera. The rotation speed of the RFS-TENG is 300 rpm and the external capacitance is 4700 pF.)

Video. S2. The illumination of the developed color-tunable SSL system before and after tribo-charging. (The video is played in normal speed (recorded by 30 fps camera of Huawei Mate20 Smartphone). The rotation speed of the RFS-TENG is 300 rpm and the external capacitance is

4700 pF.)

Video. S3. The time-variant color illuminated by the developed color-tunable SSL system for the wireless sensing application. (The video is played in 20 times slower speed (recorded by 240 fps camera of Huawei Mate 20 Smartphone). The rotation speed of the RFS-TENG is 30 rpm and the external capacitance is 12200 pF.)
